# 

**Published:** 1896-05

**Authors:** 


### CONTENTS:

### SELECTIONS.

Article I.—Photographing the Unseen. By Howard Van Rensselaer, Ph. B., M. D. - - - - - - 1
" II.—Dentistry in Japan. - - - - - - 5
" III.—Porcelain Inlays. By Dr. C. J. Essig. - - 7
" IV.—Local Anæsthesia - - - - - - 10
" V.—Electricity in Dental Practice. By Dr. L. E. Custer. 12
" VI.—Making Crowns. By Dr. C. F. Rogers. - - 15
" VII.—Specific Treatment of Necrosis of the Alveoli and Maxillæ with Aromatic Sulphuric Acid. By W. A. Mills, D. D. S. - - - - - - 17
" VIII.—Dentistry a Distinct and Independent Profession. By R. Finley Hunt, D. D. S. - - - - - 22
" IX.—The Relations of Materia Medica and Therapeutics to Stomatology. By Russell H. Cool, D. D. S. - 27
" X.—Roentgen's Discovery. - - - - - - 33
" XI.—Some Practical Points in Porcelain Work. By Dr. A. C. McAlpin. - - - - - - - 36

### EDITORIAL, ETC.

Verses by Dr. Evans. - - - - - - - - 41

### MONTHLY SUMMARY.

Dentistry in Brazil. - - - - - - - - 41

A Mechanical Horror. - - - - - - - - 43

Saving Teeth. - - - - - - - - - 43

To Sterilize Steel Instruments Without Reducing their Temper. - 44

C. E. Bentley, in Items of Interest. - - - - - 45

Relief of Headache Following Dental Operations. - - - 45

Advantages of Pyrozone. - - - - - - 46

Balsam Varnish. - - - - - - - - - 46

Filling Pulpless Teeth With Fistulous Opening. - - - 47

Spot Specialism. - - - - - - - - - 47

Administering Nitrous Oxid. - - - - - - 47

Pink Base Plate. - - - - - - - - - 48

Specific Remedies. - - - - - - - - - 48

Filling a Cavity Temporarily. - - - - - - 48

